# Supplementary material for: MetaRibo-Seq measures translation in microbiomes
Source: Nat Commun. 2020 Jun 29;11:3268. doi: 10.1038/s41467-020-17081-z (PMC7324362; doi:10.1038/s41467-020-17081-z)
Supplement: Supplementary file 10 — Supplementary Data 7 [file 41467_2020_17081_MOESM10_ESM.zip › File2/Confidence_High_Taxonomy/259051_out.krona.html]

Javascript must be enabled to view this page.

members
magnitude
magnitudeUnassigned
count
unassigned
taxon
rank

259051\_out

2

2
2
superkingdom

1239
1
phylum

class
1
186801

1
186802
order

1
186803
family

1410630

SRS893352\_contig\_number\_27975
1
species

976
1
phylum

200643
1
class

1
171549
order

1
171550
family

1
239759
genus

species
1
328814

SRS043768\_contig\_number\_24179
